# Ferret badger rabies origin and its revisited importance as potential source of rabies transmission in Southeast China

**DOI:** 10.1186/1471-2334-10-234

**Published:** 2010-08-06

**Authors:** Ye Liu, Shoufeng Zhang, Xianfu Wu, Jinghui Zhao, Yanli Hou, Fei Zhang, Andres Velasco-Villa, Charles E Rupprecht, Rongliang Hu

**Affiliations:** 1 Key Laboratory of Jilin Province for Zoonosis Prevention and Control, Laboratory of Epidemiology, Veterinary Research Institute, Academy of Military Medical Sciences, 1068 Qinglong Road, Changchun 130062, China; 2 Centers for Disease Control and Prevention, 1600 Clifton Rd, Atlanta, GA 30333, USA; 3 Nanling Campus, Jilin University, Changchun 130022, China

## Abstract

**Background:**

The frequent occurrence of ferret badger-associated human rabies cases in southeast China highlights the lack of laboratory-based surveillance and urges revisiting the potential importance of this animal in rabies transmission. To determine if the ferret badgers actually contribute to human and dog rabies cases, and the possible origin of the ferret badger-associated rabies in the region, an active rabies survey was conducted to determine the frequency of rabies infection and seroprevalence in dogs and ferret badgers.

**Methods:**

A retrospective survey on rabies epidemics was performed in Zhejiang, Jiangxi and Anhui provinces in southeast China. The brain tissues from ferret badgers and dogs were assayed by fluorescent antibody test. Rabies virus was isolated and sequenced for phylogenetic analysis. The sera from ferret badgers and dogs were titrated using rabies virus neutralizing antibodies (VNA) test.

**Results:**

The ferret badgers presented a higher percentage of rabies seroconversion than dogs did in the endemic region, reaching a maximum of 95% in the collected samples. Nine ferret badger-associated rabies viruses were isolated, sequenced, and were phylogenetically clustered as a separate group. Nucleotide sequence revealed 99.4-99.8% homology within the ferret badger isolates, and 83-89% homology to the dog isolates in the nucleoprotein and glycoprotein genes in the same rabies endemic regions.

**Conclusions:**

Our data suggest ferret badger-associated rabies has likely formed as an independent enzootic originating from dogs during the long-term rabies infestation in southeast China. The eventual role of FB rabies in public health remains unclear. However, management of ferret badger bites, rabies awareness and control in the related regions should be an immediate need.

## Background

Rabies is caused by neurotropic viruses in the genus *Lyssavirus*, family *Rhabdoviridae*, and is transmissible to all mammals [1]. Dogs are the main hosts responsible for human rabies in Africa, Latin Americas and Asia, especially in China [2,3], where rabies is re-emerging as a major public health threat, and its severity is only second to HIV and tuberculosis (TB) among all reportable infectious diseases. From the annual ~3000 human deaths, southeast China counts for most cases, with more than 90% attributed to rabid dog bites [3]. Notably, both human population and dog density are high in the region with low rabies vaccination coverage in dogs. Given that the program of dog rabies elimination has not been listed in the priority of governmental agenda, it is possible that long term dog rabies enzootics will lead to spillover events of dog-associated rabies into wildlife species. In addition to rabies transmitted by rabid dogs, other sources of rabies exposure to humans, such as cats, ferret badgers (FB), and pigs, have been continuously reported in China [4-8]. Interestingly, in provinces like Zhejiang, Jiangxi and Anhui, the percentage of dog-associated human rabies is relatively low. Meanwhile, up to 80% of the reported human rabies cases were inferred to be caused by FB bites in some districts in Zhejiang province from 1994 to 2004 [9]. Although rabies in badgers was previously recorded in other countries [10,11], FB-associated human rabies has never been reported except in China [12,13]. The frequent occurrence of FB-associated human rabies cases in southeast China highlights the lack of laboratory-based surveillance and urges revisiting the potential importance of this animal in rabies transmission. Nevertheless, management of such animal bites in humans needs a clear guideline on post-exposure prophylaxis (PEP) for rabies. Currently, FB trading and its meat consumption are common in the related areas, resulting in a frequent source of FB bite to humans. Similar to severe acute respiratory syndrome (SARS) outbreaks through consumption of civet in south china, the close and frequent contact of FB by humans could be an important factor in human rabies cases in southeast China.

To determine if the FB actually contributes to human and dog rabies cases, and the possible origin of the FB-associated rabies in the region, we conducted an expanded retrospective/prospective epidemiological survey, which encompassed both descriptive and molecular epidemiological approaches.

## Methods

### Epidemiological survey on FB-associated human rabies cases

A retrospective survey on rabies epidemics was carried out in Zhejiang, Jiangxi and Anhui provinces in southeast China. The data were collected and summarized from the provincial CDC surveillance system and epidemiological records. Some information was obtained verbally after interviewing animal traders or hunters in the endemic areas. In human rabies cases and its potential association with FB transmission, we conducted a preliminary investigation of FB population density, exposure frequency of sick FB to humans, and management of rabies PEP after a FB bite.

### FB and dog brain specimens and Fluorescent Antibody Test (FAT)

Dead or live FB collected in fields and houses, and dogs that had bitten people were sent by selected farmers to the designated laboratories for diagnoses. The surveyed mountainous areas were Huzhou, Hangzhou, Jinhua, Quzhou, Lishui in Zhejiang province; Nanchang, Jingdezhen, Shangrao, Wuyuan in Jiangxi province; and Huangshan, Xuancheng, Anqing, Jingxian in Anhui province. The animal heads were packed appropriately and shipped to our laboratory under cold conditions. The brains were removed by opening the skull under sterile conditions.

Animal experiments related to this study were approved by the Committee of Animal Welfare and Ethics of Veterinary Research Institute, Academy of Military Medical Sciences. Humane endpoints were used throughout this study in accordance with the ethical principles for in vivo studies. All animals including ferret badgers, dogs and mice that showed clinical signs of infection were killed humanely. This study did not refer to any issue of human ethics as only the epidemiological data were collected and analyzed, and no sample was collected from any healthy and contracted humans.

The FAT on brain specimens was performed according to methods described elsewhere [14]. Briefly, the brain tissue impressions were made on slides. After air drying, the slides were fixed with 80% acetone for 20 min. The FITC-conjugated anti-rabies virus nucleoprotein monoclonal antibodies (made in Laboratory of Epidemiology, Veterinary Institute, Changchun, for routine rabies diagnosis) was added and incubated for 40 min at 37ºC. After three-times' wash using PBS buffer (0.01 M, pH 7.4) -Tween-20 (0.1%), the slides were left to dry in the air at ambient temperature, and observed under a UV fluorescent microscope (Zeiss Corporation, Germany) for typical rabies virus staining.

### Mouse inoculation test (MIT)

For FAT positive specimens, the suspension of the sample was injected intracerebrally to 1-day-old suckling mice (Kunming mice, Animal Core Laboratory, Changchun Institute of Biological Products) according to protocols described elsewhere [15]. The mice were observed for ~28 days. The brain smear was made to re-check for the presence of rabies virus antigen using the FAT if the inoculated mice died from day 6 to 28.

### Sequences of the nucleoprotein and glycoprotein genes and phylogenetic analysis

Total RNA of the infected FB brains were extracted using Trizol reagent (Invitrogen, Carlsbad, CA). The nucleoprotein (N) and glycoprotein (G) genes were amplified by RT-PCR following the protocol described by Nadin-Davis et al [16] using the total RNA with the following primers. The positions of the primers were referred against strain FJ009 (GenBank *Acc. No*. FJ866836.1).

RV-N-F (1-25): 5'-ACGCTTAACAACAAAACCATAGAAG-3';

RV-N-R (1515-1538): 5'-CGGATTGACGAAGATCTTGCTCAT-3';

RV-G-F (3291-3315): 5'-CATCCCTCAAAAGACTTAAGGAAAG-3';

RV-G-R (4918-4941): 5'-CCGAGGAGATGAGGTCTTCGGGAC-3',

The TaKaRa (TaKaRa Corp Ltd, Dalian, China) was the contracted company responsible for sequencing the amplicons. We generated a phylogenetic tree using the Neighbor-joining (NJ) method in MEGA 4 (MegAlign, DNASTAR Software Suite, Version 7.1.0, Copyright 1989-2006, DNASTAR Inc.). The Bootstrap values were calculated from 1,000 repeats using 70% as the cut-off.

### Rabies virus (RV) neutralizing antibodies in dog and FB serum samples

Serum samples were collected from the captured FBs and dogs in our expanded surveyed areas. All the FBs were alive and appeared healthy when the serum was collected. Dog sera were from watchdogs belonging to residents of different villages in Zhejiang, Jiangxi and Anhui provinces. The method of titration of virus neutralizing antibodies (VNA) by Fluorescent Antibody Virus Neutralization (FAVN) test was described elsewhere [17]. Briefly, 3-fold serial dilutions of standard serum (0.5 International Units, IU/ml) and test serum samples were prepared in microplates in quadruplicate. Fifty _μl of challenge rabies virus (CVS-11) containing 100 TCID_50 _was also added to each well. After 60 min incubation at 37°C in a humidified 5% CO_2 _incubator, 50 μl cell suspension containing 2 × 10^4 ^cells was added to each well and the plates were incubated for 48 h at 37°C. After fixation at room temperature for 30 min in 80% acetone, the cell monolayers were stained by addition of FITC-conjugated anti-rabies nucleoprotein monoclonal antibodies (Laboratory of Epidemiology, Veterinary Institute, Changchun, China) to each well. Staining was carried out in an incubator at 37°C for 30 min and fluorescence was observed by UV microscope (Zeiss, Germany). Wells exhibiting no specific fluorescence were considered antibody positive. Neutralizing antibody titers were calculated using the Spearman-Kärber formula and expressed in IU/ml by comparison with a reference serum (13.5 IU/ml, product of AFSSA, France), 0.1 IU/ml is considered as the cut-off of seroconversion after rabies virus infection in both ferret badgers and dogs.

## Results

### Epidemiological survey on FB-associated human rabies cases and preliminary ecological investigation on FB population

Human rabies cases potentially associated with FB transmission were inferred retrospectively and prospectively on the basis of exposure records (Table 1). The patients included FB-hunters who capture and sell FBs, farmers with occasional exposure to sick FBs, and residents who were exposed to sick FBs in their yard or house. Healthy FBs do not actively attack humans because of their nocturnal behavior, but rabid FBs become excited, run into residential areas, and will bite. In the case of hunters or occasional hunters, exposure to FB happened almost every day. The FB bites in humans were usually on the hands, feet, or occasionally on the arms, legs, and very rarely upon the upper body.

**Table 1:** FB-associated human rabies cases inferred from exposure history

Provinces	Counties	Human cases	Period	Total human rabies cases in a province or at a region
Zhejiang	Changxing	6	1994-1995	N/A

Zhejiang	Lin'an, Chun'an, Tonglu, Jiande, Quzhou, Huzhou, Changxing	29	1996-2004	42

Zhejiang	Hangzhou	2	2006-2007	N/A

Zhejiang	Lishui	1	2008	N/A

Jiangxi	Wuyuan	4	2007-2008	5

Anhui	Xuancheng	2	2004-2005	N/A

Anhui	Shexian	2	2003-2004	N/A

Anhui	Huangshan	2	2002	N/A

Anhui	Jingxian	1	2003	N/A

N/A: Not available

The population density of FBs is unknown. Empirically, hunters lay their traps according to the number of FB tracks. On average, 2-5 tracks could be found within 80 Chinese acres (1 Chinese acre = 660 m^2^). The number of FB is about 38-95 per km^2^. However, this rough estimation needs ecological support. In our investigation, there was at least one hunter in almost every village. Seasonally, more than 1 FB was caught by a hunter per day. Despite frequent contact with rabies- susceptible animals, no hunters are aware of the potential danger of rabies exposure. No PEP occurred after FB bites. Furthermore, the hunters and farmers live in remote rural areas and mountainous regions, and their income (1000 RMB or less per month) cannot afford the high cost of PEP (1300-2000 RMB) in China. Therefore, the recommendation of PEP is not only ignored, but also intentionally neglected due to the associated costs.

### FAT and MIT

In total, we collected 209 dead FB from our surveyed regions, and 76 of 209 were partially decomposed when the brains were removed for diagnosis. Eight of 209 brain samples were confirmed rabies positive using FAT (3.8%), and RV was successfully isolated by MIT from the samples. The rabies incubation period in 1-day-old suckling mice was 7-12 days.

From the 56 brain samples we collected from live FBs in Jiangxi, Anhui and Zhejiang provinces, only 1 sample was rabies positive by FAT (1.8%, 1/56). Since captured and injured by the hunter, this positive animal did not present obvious clinical signs of rabies before euthanized for diagnosis. From the 15 dog brain samples collected in the same regions, 2 were rabies positive by FAT (13%, 2/15). All RVs from FAT positive samples were successfully isolated by MIT.

### Rabies VNA in FB and dog serum samples

Fifty six serum samples were collected from live FBs in the selected 3 provinces. The average rabies seroconversion rate was 69.6%, ranging from 0 (no neutralizing antibody in all individuals) to 95% in different collections, and rabies VNA ranged from 0 to 2.6 IU/ml (Table 2). In the 77 dog serum samples, the detectable VNA was 18.2% (14/77), and the overall percentage of VNA positive was relatively lower from dogs than the samples from FBs (Table 3). Since no rabies vaccination campaign has been performed in the FBs and dogs could be vaccinated occasionally due to the disease awareness by the owners, the higher seroconversion in the FBs is an interesting phenomenon.

**Table 2:** Rabies virus neutralizing antibodies in ferret badgers

Origin places	Specimen numbers and serum VNA levels (IU/ml)									
	
	1	2	3	4	5	6	7	8	9	10
Jingdezhen, JX	0.66	0.22	0.50	0.66	0.22	0.50	1.14	0.22	0.66	1.97

Shashi, JX	0.22	0.66	0.22	0.22	0.50	0.50	0.66	1.97	0.50	2.6

Lin'an, ZJ	0.22	0.10	0.22	0*	0.17	0.22	0.22	0.22	0.22	0.22

Chun'an, ZJ	0.22	0.29	0	0.22	0.22	0.17	0.29	0.66	0.29	0.87

Quzhou, ZJ	0	0	0	0.22	0	0	0	0	0	0

Huzhou, ZJ	0	0	0	0	0	0	/	/	/	/

Note: / means not available. JX: Jiangxi province; ZJ: Zhejiang province.

*A rabies virus was isolated from the brain tissue of this ferret badger.

**Table 3:** Rabies virus neutralizing antibodies in dogs

Origin places	Specimen numbers and serum VNA levels (IU/ml)												
	
	1	2	3	4	5	6	7	8	9	10	11	12	13
Jingdezhen, JX	0	0.97	0	0	0	0	0	0	0	2.17	0	0	0

Xuancheng, AH	0	0	0	0	0	0	0.50	0	0	0	0	0	0

Lin'an, ZJ	0.65	0	0	0	0	1.17	0	0	0	0.22	4.5	0	0

Chun'an, ZJ	0	9.3	0	0	0	2.12	0	0	0	0	0	0	0

Quzhou, ZJ	1.17	0	0	0.22	0	0	11.3	0	0	0	0	0	0

Huzhou, ZJ	0	0	0	0	0.33	0	0	1.50	0	0	0	0	/

Note: / means not available. JX: Jiangxi province; AH: Anhui province; ZJ: Zhejiang province.

### Sequence and phylogenetic analysis

**Figure 1:**
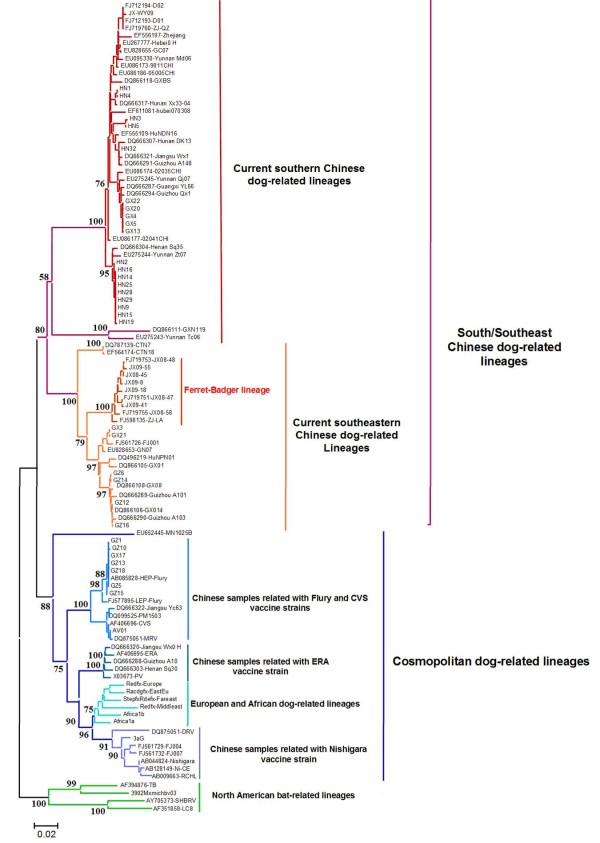
**The neighbor-joining phylogenetic tree of Chinese FB rabies virus isolates, using the full length N gene (1353 bp) for reconstruction**.

We compared RV N sequences from rabid FB isolates with those in dogs from China, foxes from Europe/Middle East, dogs in Africa, and rabies vaccine strains from Asia, Europe and the US (Figure 1). Dog RVs in China were categorized into two groups. Group 1 compromises RVs all across south and southeast China, and group 2 is formed by Chinese isolates distributed in Guizhou, Jiangsu, Henan, Jilin provinces, and is closely related to Asian and occidental vaccine strains, and cosmopolitan dog RVs (Figure 1). In general, Chinese dog-associated lineages shared nucleotide identity from 87 to 98% (Figure 1). Same phylogenetic pattern was reconstructed using the G gene sequences (data not shown).

The FB RVs were segregated in an independent lineage (Figures 1). The nucleotide identity of FB isolates with current south and southeast Chinese dog-related lineages (from Zhejiang, Jiangxi, Fujian, Jiangsu, Hunan, Guangxi, Guizhou, Hubei, Yunnan, Henan) ranged from 87 to 89% (94.5-98.8% homology by amino acid sequence comparison). However, the intra-group identity in the 9 FB isolates only varied from 98.7 to 99.1% (99.0-100% homology by amino acid sequences). A notable difference was detected on the G protein motif at residual position 331-333 with Serine-Valine-Arginine (S-V-R) in FB isolates. This motif has not been found in any isolates from dogs in China and the corresponding motif in dog isolates was Serine-Isoleucine-Arginine (S-I-R), which does not exist in the FB isolates (using PV as the reference sequence, GenBank *Acc. No*. M13215).

## Discussion

The current reporting system of human rabies in China covers two aspects. One is the statistical distribution status of human rabies cases, which can be reviewed on governmental websites by authorized personnel only. The other is the documented epidemiological data of each case available at local provincial CDCs. The establishment of this system occurred after the lessons learned from the SARS outbreaks. Although annual epidemiological data represented about 3,000 human rabies deaths during the last five consecutive years, rabies is still not considered as a priority in the China public health system.

Although FB-associated human rabies cases have been reported in China [6,18,19], the actual number of human cases remain unknown. In Africa, about 0.33% rabies seroconversion was recorded in badger populations [10]. Rabies in such animals was detected in Africa [10] and Europe [11], but was simulated in Great Britain [20]. However, FB-associated human rabies has never been reported except in southeast China. In our surveyed counties, where 69-80% of human rabies cases were inferred to be caused by this animal species, such epidemiological data lack laboratory-based diagnosis, and may confuse the actual role of FB in the transmission of rabies to domestic dogs and humans. Therefore, in this study, we conducted an active laboratory-based rabies survey to determine the frequency of rabies virus infection and seroprevalence in both dogs and FBs. The percentage of RV infection was 3.8% (8/209) in dead FBs, 1.8% (1/56) in live FBs and up to 13% (2/15) in domestic dogs that had bitten people. Conversely, apparently healthy FBs were found with a high percentage of rabies seroconversion (69.6%), whereas dogs were only 18.2%. However, no rabies vaccination program has been performed in wildlife in China. The high percentage of rabies seroconversion in FBs is an interesting discovery, and could be due to abortive rabies infections in the population. Domestic dogs still are the most affected species, and are the most likely source for rabies transmission to humans and other animals in the endemic region. Our data also suggest that FBs may not be as susceptible to rabies as other carnivores.

In the 77 dog serum samples, the detectable VNA was 18.2% (14/77), and the overall percentage of VNA positive was relatively lower than the samples from FBs. However, some of the VNA positive dogs had high levels of antibodies with two reached 9.27 and 11.3 IU/ml, respectively. We think the randomly collected serum samples include those from vaccinated dogs due to rabies awareness by the owners.

The consistent amino acid signatures along with the phylogenetic findings in this report suggest that the FB RV variant may be maintained as an independent enzootic by local FB populations. Nonetheless, the origin of FB RV has its root in south/southeast China dog RV variants. Our phylogenetic analysis indicates a broad co-circulation of at least two major dog-related groups throughout south/southeast China. Group 1 is relatively distant to the FB lineage, and seems to act as a new emergence. Group 2 is closely related to the FB lineage, and connects to historical dog RVs circulating in the late 1950s in China. As human populations in southeastern China are condensed and widely distributed in hills and mountainous area, their contact with rabid FBs could become a greater potential source of exposure to RV. In some rabies endemic regions, illegal FB trade for meat consumption increased the risks among hunters, farmers, traders, slaughterers, and chefs. The awareness of rabies should be a priority in the southeast populations at risk. Clearly PEP should be initiated after FB bite, provided that timely post-mortem rabies diagnosis of the biting animal is not feasible.

For eventual rabies control, China should implement more effective rabies surveillance programs structured by animal control units (where suspicious animals are seized, euthanized and sampled) and a highly proficient rabies diagnostics laboratory network at the governmental and local levels to detect and characterize any rabies outbreaks in any susceptible hosts. The circulation of a dog-related RV variant in wildlife populations (in this case of FB) may pose a severe delay and complicate the elimination of dog and human rabies. With a low vaccination coverage and high dog population density, the FB RV may potentially return to the dog population, or vice versa. Thus, an integral rabies control program should be implemented, targeting both dog and FB populations, by using novel vaccination strategies. Otherwise, the goal of controlling animal rabies and eliminating human rabies by 2020 may not be achievable (the aim set by ASEAN plus 3 rabies conference).

## Conclusions

In conclusion, we demonstrated that FB rabies is likely occurring as an independent enzootic that became established in the FB populations from a dog RV variant distributed in southeast/south China. The actual role of FB rabies in public health remains unclear. However, since potential rabies transmission from FB to humans and dogs cannot be excluded, immediate rabies awareness should be altered. Standard PEP should be recommended once an exposure is confirmed by reliable laboratory diagnostics. To meet the goal of elimination of rabies in humans by 2020, China must strengthen its rabies surveillance system and develop feasible strategies and programs for vaccination of dogs and wildlife.

## Competing interests

The authors declare that they have no competing interests.

## Authors' contributions

YL carried out the epidemiological survey on ferret badger-associated human rabies. SZ and XW carried out the sequence alignments and analysis on phylogeny. JZ participated in specimen collection of both ferret badgers and dogs. YH participated in the sequence analysis using MEGA4/MegAlign softare, AVV analyze the results of molecular epidemiology, FZ assayed the viral neutralizing antibody, CR and RH designed the study, wrote the manuscript and coordinated the research. YL, SZ, XW and JZ contribute equally to this manuscript. All authors read and approved the final manuscript.

## Pre-publication history

The pre-publication history for this paper can be accessed here:

http://www.biomedcentral.com/1471-2334/10/234/prepub
